# Development and validation of Health Belief Model based instrument to assess secondary school student’s adherence to COVID-19 self-protective practices in Jimma, Oromia, Ethiopia

**DOI:** 10.1371/journal.pone.0279440

**Published:** 2022-12-21

**Authors:** Kasahun Girma Tareke, Genzebie Tesfaye, Zewdie Birhanu Koricha

**Affiliations:** Faculty of Public Health, Department of Health, Behavior and Society, Institute of Health, Jimma University, Jimma, Ethiopia; Nazarbayev University School of Medicine, KAZAKHSTAN

## Abstract

**Background:**

The study aimed in developing and validating a Health Belief Model (HBM) based instrument used for cross-sectional studies among secondary school students in Jimma town, Oromia, Ethiopia.

**Methods:**

A school-based cross-sectional study was conducted from May 25 to June 10, 2021. The sample size was 634, and students were randomly selected from public and private secondary schools. The 81 items were developed reviewing different literatures based on the constructs of HBM. The constructs were perceived severity, perceived vulnerability, perceived benefit, perceived barrier, self-efficacy, cues to action, perceived school support and self-protective practice. Data were collected using a self-administered questionnaire. The data were cleaned, entered into and analyzed using SPSS 23.0. A principal axis factoring with varimax rotation was carried out to extract items. Items with no loading factor or cross-loaded items were deleted. Items having factor loading coefficient of ≥0.4 were retained. An internal reliability was ensured at Cronbach’s alpha >0.70. All items with corrected item-total correlation coefficient below 0.30 were deleted from reliability analysis.

**Results:**

In this study, 576 respondents were participated making a response rate of 90.8%. A total of thirty items were extracted and loaded in to eleven factors with cumulative variance of 56.719%. Percieved social support, percieved benefit, percieved school responsibility, self-efficacy, and practice items were internally consistent. Percieved vurnerability was neither valid nor reliable construct. Similarly, from the extracted factors, attitude towards face mask use and percieved peer influence were not internally consistent. Lastly, percieved benefit, self-efficacy and percieved school responsibility significantly predicted student’s adherence to COVID-19 self-protective practices.

**Conclusions:**

The study found that perceived benefit, perceived school support, social support, self-efficacy, perceived school environment cleanness, perceived school responsibility, perceived school health education, attitude to use face mask, perceived severity, cues to action and perceived peer influence were valid. Finally, perceived benefit, self-efficacy and perceived school responsibility significantly predicted student’s adherence to COVID-19 self-protective practices.

## Background

The coronavirus (COVID-19) declared as a global pandemic disease on 11 March 2020. In response, countries of the world, including Ethiopia adopted different prevention methods and informed or ordered the community to practice of safety and preventive measures such as maintaining physical distancing, use of face mask, lock down, hand hygiene, and avoid touching the eyes, nose, and mouth before washing hands, avoidance of crowded places, movement restriction, banned social gatherings, promotion of frequent hand washing and respiratory hygiene, quarantine of international travelers, and declaration of emergency state [1–7].

In addition, closure of school (all lower level and higher institutions) was also made as a safety measures to prevent and stop the spread of the virus as schools are the commonest places where peoples/students are overcrowded to facilitate and increase risk of transmission and spread of the disease [2,3,8]. Thus, the pandemic disturbed the normal flow of academic progress in different countries and also facilitated school absenteeism [9]. For example, in Ethiopia, more than 26 million students were affected in Ethiopia [10].

However, schools were re-opened after evaluating the nature of COVID-19 disease as it is likely to stay over an extended period. Greater emphasis was given while reopening the schools and the school community, including students to adhere to safety measures such as maintaining physical distance both in the classroom and outside the classroom; reducing student size per class; consistent use of face mask, frequent hand washing with soap and water; regular screening for symptoms of COVID-19, provision of regular health education, cleaning and disinfection, adequate ventilation, spacing of desks or grouping of children if required and availability necessary resources [2,7,8,10].

This is because it is a place where highest segment of the population spends time. So, School community would act as a potential amplifier of the COVID-19 transmission and speedup its spread both for the school and general community. Therefore, practicing self-preventive practices at school is essential to prevent transmission or reduce spread of the infection [2,10,11]. Moreover, it is crucial to make students have readiness appropriate knowledge about the diseases; make them perceive about the risks and severity for the infection, and practice safety and preventive measures [7,8,10].

However, as per the author’s best knowledge, little is known regarding students’ adherence to the COVID-19 prevention measures is being stayed a certain periods after the schools were reopened. Furthermore, for better implementation of COVID 19 prevention measures at schools, it is critical to assess the students’ self-protective practices. Moreover, as there is no existing evidence indicated a validated tool to assess the self-preventive practices, this study mainly aimed in developing and validating an instrument used to assess COVID-19 self-protective practice, and its associated factors among secondary school students.

The instrument was developed based on the constructs of Health Belief Model (HBM), which is one of the most commonly used behavioral models given that adherence to self-protective practice is a key behavior in preventing the transmission or spread of COVID-19 infection. It proposes that preventive health behavior is influenced by five factors: perceived barriers to making the recommended response, perceived benefits of making the response, perceived susceptibility to the health threat, perceived severity of the threat, and cues to action [12]. In addition, perceived school support was also included in the tool. This is because; schools were reopened considering school environment need to be supportive for school community to prevent COVID-19 preventive practices. Also, schools are infrastructures where large segment of the population, especially the youth and adolescents spent more of their time and interact with each other. Therefore, there is a need to include this context-based dimension as it has its contribution in preventing or dissemination COVID-19 infection [10,12,13].

Thus, development and validation of the tool was focused on the perceived severity, perceived vulnerability, perceived benefit, perceived barrier, cues to action, self-efficacy, self-protective practice and perceived school support. Reliability analysis and tool validation an essential criterion for evaluating the quality and acceptability of research as it explains how well the collected data covers the actual area of investigation. Furthermore, the quality of an instrument is very critical because the conclusions researchers draw are based on the information they obtain using specific instruments. Validity has many types which includes face validity (i.e., subjective judgment of defined constructs), content validity (i.e., the degree to which items in an instrument reflect the content universe to which the instrument will be generalized through review of literature followed by evaluation by expert judges or panels), construct validity (i.e., how well you translated or transformed a concept, idea, or behavior that is a construct into a functioning and operating reality, the operationalization), and criterion validity (i.e., the extent to which a measure predicts an outcome) [14–18]. Therefore, this study describes the processes undergone to develop and validate (i.e., construct and predictive validity) the instrument developed based on the constructs of HBM. The validated tool would be useful for any researchers who will conduct cross-sectional studies on similar research areas.

## Methods and materials

### Study design, setting and period

A school-based cross-sectional was conducted in Jimma town, Jimma, Oromia regional state, Ethiopia, from May 25 to June 10, 2021. Jimma town is the largest city in southwestern Oromia, and located 350 km away from the Addis Ababa. According to the 2007 population census, the town had an estimated total population of 159,009; of whom 80,897 were males and 78,112 were females [19]. In the town, there were 14 secondary schools (8 private and 6 public schools) with a total number of 10,720 students [20]. Secondary school students (both public and private) enrolled into grade 9–12 in the regular program during the 2021 academic year were study populations.

### Procedures and phases of instrument development and validation

#### Step-one: Review of existing literatures and generation of items

Reviewing different literatures were reviewed [10,21–24], a total of 81 items (i.e., 67 for independent variables and 14 for dependent variable) were generated [S1 File]. The items aligned to the theory of HBM constructs. A 3-response scale was used to measure the constructs/dimensions. This is because, existing evidences recommend it given that a five response rate difficult for the study participants to easly estimate the level of difference in between the scales. We also recognized such problems while conducting a pretest. Thus, for COVID-19 self-protective practices, 14 items were generated on rating scale as always (3), sometimes (2), and never (1) for desirable healthy practices relevant for COVID-19 prevention and safety considering the last seven days before the survey [10,21–24]. The perceived vulnerability and severity was assessed using seven items per each construct on three points scale (Disagree = 0; not sure = 1 and agree = 2). Perceived self-efficacy was measured using seven items rated as low (1), moderate (2), and high (3).

Perceived benefit and perceived barrier was measured by using nine and thirteen questions, respectively with a three-point scale rated as, 0 = Disagree, 1 = not sure, and 2 = Agree. On the other hand, perceived school support (the perceptions that the school environment is safe to protect oneself from COVID-19) [12,13] was measured to assess the extent to which students feel that necessary support facilities and resources are readily available to them and perceive that the school environment is safe to them [10]. A total of 20 items were developed under this dimension using a rating scale as always (= 3), sometimes (= 2), and never (= 1). Four items were developed to measure cues to action was measured by using four items with “yes, and no” response options.

#### Step-two: Scale development

Next to the generation of items, face and content validity was conducted with through consulting experts in behavioral models. Seven academicians who have research experience on behavioral models were consulted to provide their subjective judgment on the items and operationalization of a construct following indicators such as representativeness, redundancy of items, clarity, and relevance… First, we approached them through email and get feedback. Then, phone calling and physical contact was done with them for feedbacks needing elaboration or clarity. All necessary revisions were made based on the expert’s opinion. In addition, the items were generated reviewing different literatures to ensure content validity [14,17,18]. Moreover, the instrument was pretested on 5% of the sample in secondary school similar contexts and amendments were done to improve clarity, understandability and quality of tool.

#### Step-3: Data collection

Data were collected from a total of 634 sampled secondary school students. Both public and private school students were participated on the study. Half of the public schools (3 out of 6) and 3 of the private schools (3 out of 8) were randomly selected from each cluster. Following this, the total sample size was proportionally allocated to each selected school based on student size. Within each selected school, the further proportional allocation was done by grade levels. Finally, an updated list of students was obtained from each grade and the actual study participants were selected using a simple random sampling technique. The data were collected through the self-administrated method, assisted by data collectors and coordinators in each school. Adequate orientation, instructions, and guidance were provided to study participants by data collectors.

#### Step-4: Statistical analysis

The collected data were checked completeness and cleaned to ensure quality, accuracy, consistency. Then, it entered and analyzed using the Statistical Package for Social Science (SPSS) software version 23.0. A confirmatory factor analysis (CFA) was tried to test validity of existing factors using a structural equation modeling using Stata version 14 software. However, due to a need of too many modification to fix the model, it was left and an exploratory factor analysis was done [25,26]. An exploratory factor analysis using principal axis factoring (PAF) with varimax rotation was carried out to extract the high loading items and evaluate the construct validity of the scale. Items with no loading factor or cross-loaded items were deleted and only items having factor loading coefficient of ≥0.4 were retained [14–18]. Appropriateness or significance of the factor loading test was ensured Bartlett’s Test of Spherity and Kaiser-Meyer-Olkin (KMO). Similarly, an Eigenvalue of greater than 1 was used as a cut-off point. All items with inter-item and item-total correlation coefficient value below 0.30 were removed from further analysis. A Cronbach’s alpha of greater than or equal to 0.7 was used as the cutoff point establishes reliability of the scale [14–18]. Finally, linear regression analysis was done to ensure predictive validity of the items.

### Measurements

#### COVID-19 self-care practices

Self-care practice (refers to use of recommended COVID-19 self-protective and safety measures in school context) [21–24] was assessed in a comprehensive way using fourteen items on rating scale as always (3), sometimes (2), and never (1) for desirable healthy practices relevant for COVID-19 prevention and safety considering the last seven days before the survey.

#### Perceived vulnerability and severity

The perceived vulnerability (refers to one’s perception of the risk or the chances of contracting a COVID-19) [12,24] was assessed using seven items on three points scale (Disagree = 0; not sure = 1 and agree = 2) and perceived severity (refers to an individual’s belief about the seriousness of contracting COVID-19 or the severity of the consequences if one acquire it) was assessed using seven items on a similar rating scale.

#### Perceived self-efficacy

Perceived self-efficacy (refers to the level of a person’s confidence in his or her ability to successfully perform a behavior) [12,24] to exercise COVID-19 protective measures was measured using seven items rated as low (1), moderate (2), and high (3).

#### Perceived school support/safety

Perceived school safety (the perceptions that the school environment is safe to protect oneself from COVID-19) [10,12,13,24] measure has assessed the extent to which students feel that necessary support facilities and resources are readily available to them and perceive that the school environment is safe to them [12]. A total of 20 items was administrated to the respondents using a rating scale as always (= 3), sometimes (= 2), and never (= 1).

#### Perceived barriers

Perceived barriers (refers to a person’s feelings on the obstacles to performing a recommended health action) [12,24] were measured by using thirteen questions with a three-point scale rated as 0 = disagree, 1 = not sure, and 2 = Agree.

#### Perceived benefits

Perceived benefits (the desire to avoid illness and the belief that a behavior can prevent the illness) [12,24] were measured by using nine questions with a three-point scale rated as, 0 = Disagree, 1 = not sure, and 2 = Agree.

#### Cues to actions

Cues to action was measured by using three items with “yes, and no” response options [12]. Accordingly, the probable sum score of cues to actions for the COVID-19 prevention measure was ranged from 0 to 3.

Finally, for each of the constructs/dimensions independently, all the items were summed up yielding a probable sum score of ranged from 0 to 50. Previous studies informed that the items of each constructs are summed up and rescaled to (0–100) value for standardization and comparison of the scales using Y = (X−Xmin)/Xrange *n, where Y is the adjusted variable, X is the original variable, Xmin is the minimum observed value on the original variable and Xrange is the difference between the maximum score and the minimum score on the original variable and n is the upper limit of the rescaled variable. Therefore, first, we computed a separate composite score for each dimension and construct and the mean value was calculated from the composite score separately after the score adjusted to 50% [24]. The higher score indicating the higher comprehensive self-care practices and vice versa.

### Ethical considerations

The study protocol was received and approved by an ethics review committee of institute of health, Jimma University. Permission was obtained from each school included in the study and Jimma town education office. A written informed consent was obtained from each study participant thoroughly explaining the objectives and purpose of a study. Additionally, written consent was also taken from parents of those participants whose age was under 18 years. All COVID-19 preventive measures were applied during data collection.

## Results

### Socio-demographic characteristics of participants

Five hundred seventy-six respondents participated in this study, which made a response rate of 90.8%. There was no missing data. About 296 (51.4%) of participants were female. The age of the participants ranged from 14–22 years old, with a mean age of 16.3 (SD± 1.4) years. Regarding religion, the majority 225 (39.1%) them were orthodox. Almost all 538 (93.4%) participants were single. About 522 (90.6%) of students were from public schools. Regarding the educational status, the majority 139 (24.1%) of the students’ mothers attended primary school. Similarly, nearly one-fourth of participants’ fathers had attended secondary school (Table 1).

**Table 1:** Socio-demographic characteristics of the study participants, Jimma town, Oromia, Ethiopia, 2021.

Variable	Categories	Frequency	Percent
**Sex**	Male	280	48.6
	Female	296	51.4
**Age**	14–18 years	460	79.9
	19–22 years	116	20.1
**Religion**	Orthodox	225	39.1
	Muslims	209	36.3
	Others (Protest, catholic)	142	24.7
**Marital status**	Single	538	93.4
	Others (Divorce, widowed	38	6.6
**School type**	Private	54	9.4
	Public	522	90.6
**Grade level**	9.00	185	32.1
	10.00	150	26.0
	11.00	136	23.6
	12.00	105	18.2
**Mothers’ occupation**	Government employ	123	21.4
	Private employ	138	24.0
	Housewife	246	42.7
	Famers	50	8.7
	Others (not alive)	19	3.3
**Fathers’ occupation**	Government employ	194	33.7
	Private	247	42.9
	daily laborer	22	3.8
	Famers	67	11.6
	Others (alive,)	46	8.0
**Mother education**	No formal education	113	19.6
	Primary (1–6)	139	24.1
	Junior secondary (7–8)	119	20.7
	Secondary school (9–12)	114	19.8
	University degree and above	91	15.8
**Fathers’ educations**	No formal education	55	9.5
	Primary (1–6)	88	15.3
	Junior secondary (7–8)	89	15.5
	Secondary school (9–12)	139	24.1
	Technical vocation/diploma	113	19.6

### Factor analysis: Testing validity of the instrument

#### Explanatory factor analysis

**Fig 1:**
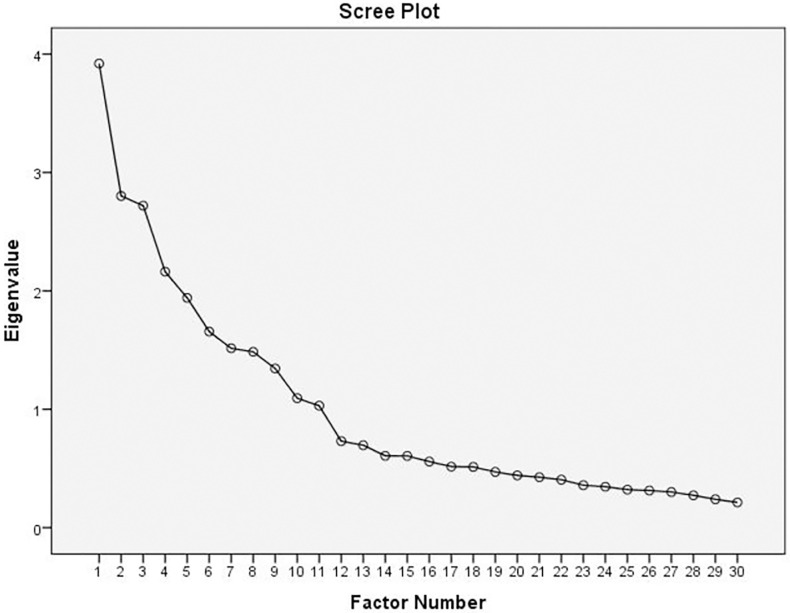


The principal axis factoring analysis (PAF) extracted thirty items under eleven factors from a total of sixty-seven items. The initial Kaiser-Meyer-Olkin (KMO) measure of the sample adequacy, indicates the appropriateness of the data for factor analysis, was 0.746 with t Bartlett’s Test of Spherity of X2 = 6506.841; df = 465 and p = 0.000. The analysis repeated many times removing items failed to load on any dimensions or loaded onto a factor other than its underlying factor (cross-loading). The final KMO was 0.678 with the Bartlett’s Test of Sphericity (X^2^ = 5697.432; df = 435 and p<0.001). The highest and lowest communality coefficient was 0.722 and 0.364 [Table 2]. The extracted items were loaded in to eleven factors with cumulative variance of 56.719% (Table 3). The extracted items were also indicated on the Scree plot (Fig 1).

**Table 2:** Communalities and factor loadings explained by extracted items among secondary school students, Jimma, Oromia, Ethiopia, 2021 (n = 576).

Rotated Factor Matrix												Communalities
Items	Factors											
	Perceived benefit	Perceived social support	Perceived school support	Self-efficacy	Perceived school responsibility	Perceived peer influence	Attitude to use facemask	Cues to action	Perceived severity	Perceived school env’t cleanness	Perceived school health education	
BEN6: I believe that avoiding from overcrowding place is helpful for me to prevent myself from COVID-19	.753											.613
BEN7: I believe that stop shaking people’s hand is helpful for me to prevent myself from COVID-19	.712											.597
BEN4: When I use facemask I feel a sense of responsibility to protect my families and communities	.668											.576
BAR13: There is no anyone motivate me to wash my hands regularly.		.828										.706
BAR14: There is no anyone motivate me to keep physical distance		.811										.725
BAR12: There is no anyone motivate me to wear a facemask		.530										.464
SS9: Signs to remind students to practice regular hand hygiene and good cough etiquette			.752									.649
SS10: Learning spaces are arranged to maximize the space available and to minimize people directly facing one another			.708									.627
SS5: Visual cues (floor markings, posters, etc.) are in place to promote physical distancing			.550									.580
SE4: How much you are confident to covering your cough/sneezing using the bend of your elbow or a tissue prevent spread of coronavirus?				.643								.504
SE3: How much you are confident avoiding touching eyes, nose and mouth prevent infection with coronavirus?				.588								.436
SE6: I can maintain at least 2 meter distance between yourself and other student to prevent infection with coronavirus				.569								.509
SE2: How much you are confident maintaining social distancing can prevent infection with coronavirus?				.496								.496
SE1: How much you are confident to washing hands frequently with soap and water or using alcohol-based hand rub kills the virus that causes COVID-19				.479								.555
SS15: Parents and students are made aware of their responsibilities in COVID prevention					.694							.572
SS14: There are an active daily Health Check for students					.673							.596
SS11: My school give attention to practice of precautionary measures for the COVID-19 pandemic in the school					.507							546
BAR9: I cannot keep physical distancing because my school is crowed						.642						.485
BAR8: I cannot stop shaking hands because my relationships with people become affected						.575						.469
BAR10: I would feel disappointed by my friends for wearing facemask						.573						.438
BAR2: Wearing facemask is unnecessary							.700					.566
BAR5: Wearing a face mask makes me look unattractive							.647					.523
CUE3: Did any of your relative or family members acquired corona virus?								.855				.781
CUE2: Have you ever seen a person who gets sick from corona virus?								.740				.568
SEV2: Do you think that COVID-19 is a dangerous disease?									.801			.675
SEV1: Do you believe that COVID-19 infection is severe disease?									.764			.617
SS13: The school’s ventilation system is serviced and operating well.										.757		.610
SS12: General cleaning and disinfecting is done every day										.698		.602
SS19: There is educational material at school to guide students practice COVID-19 preventive measures.											.678	.526
SS20: There is health education at school on COVID-19 preventive measures											.665	.506

Extraction Method: Principal Axis Factoring; Rotation Method: Varimax with Kaiser Normalization.

**Table 3:** The Eigenvalues and total variance explained by the extracted items among secondary school students, Jimma, Oromia, Ethiopia, 2021 (n = 576).

Total Variance Explained									
Factor	Initial Eigenvalues			Extraction Sums of Squared Loadings			Rotation Sums of Squared Loadings		
	Total	% of Variance	Cumulative %	Total	% of Variance	Cumulative %	Total	% of Variance	Cumulative %
1	3.920	13.068	13.068	3.497	11.655	11.655	1.932	6.441	6.441
2	2.801	9.338	22.406	2.370	7.900	19.556	1.877	6.256	12.697
3	2.720	9.066	31.472	2.287	7.622	27.178	1.825	6.085	18.782
4	2.162	7.208	38.680	1.735	5.783	32.960	1.683	5.609	24.390
5	1.941	6.470	45.149	1.539	5.131	38.091	1.555	5.185	29.575
6	1.656	5.522	50.671	1.266	4.219	42.309	1.484	4.946	34.521
7	1.515	5.051	55.722	1.115	3.716	46.025	1.453	4.844	39.365
8	1.485	4.951	60.672	1.062	3.541	49.566	1.414	4.713	44.078
9	1.344	4.481	65.153	.892	2.975	52.541	1.330	4.434	48.512
10	1.094	3.646	68.799	.662	2.206	54.747	1.279	4.265	52.776
11	1.030	3.433	72.232	.592	1.972	56.719	1.183	3.943	56.719

### Internal reliability of the instrument

Reliability of the instrument was analyzed through applying the Cronbach’s alpha efficient. The perceived severity items had a correlation coefficient of 0.622. Similarly, items of school health education, school environment cleanness, cues to action and perceived school support had 0.487, 0.532, 0.631 and 0.599 inter-item and corrected item-total correlation coefficients, respectively. On the other hand, items of perceived social support, benefit and school responsibility was internally consistent with Cronbach’s alpha value 0.778, 0.775 and 0.721, respectively. Similarly, items of self-efficacy had Cronbach’s alpha value 0.709, respectively [Table 4]. Items of used to assess adherence to self-protective practices had Cronbach’s alpha of 0.849 [Table 5].

**Table 4:** Inter-item correlation coefficient and item-total statistics of items of extracted factors used to determine adherence to COVID-19 self-protective practices among high school students in Jimma, Oromia, Ethiopia, 2021.

**Perceived severity (PS)**								
**Inter-Item Correlations**			**Item-Total Statistics**					Cronbach’s Alpha
Items	SEV1	SEV2	Scale Mean if Item Deleted	Scale Variance if Item Deleted	Corrected Item-Total Correlation	Squared Multiple Correlation	Cronbach’s Alpha if Item Deleted	
SEV1			1.9045	.142	.622	.386	.	N/A
SEV2	.622		1.8576	.220	.622	.386	.	
**Perceived school health education (PSHE)**								
**Inter-Item Correlation**			**Item-Total Statistics**					Cronbach’s Alpha
Items	SS19	SS20	Scale Mean if Item Deleted	Scale Variance if Item Deleted	Corrected Item-Total Correlation	Squared Multiple Correlation	Cronbach’s Alpha if Item Deleted	
SS19			1.9740	.547	.487	.237	.	N/A
SS20	.487		1.9740	.679	.487	.237	.	
**Perceived school environment cleanness (PSEC)**								
**Inter-Item Correlation**			**Item-Total Statistics**					Cronbach’s Alpha
Items	SS12	SS13	Scale Mean if Item Deleted	Scale Variance if Item Deleted	Corrected Item-Total Correlation	Squared Multiple Correlation	Cronbach’s Alpha if Item Deleted	
SS12			1.2222	.316	.532	.283	.	N/A
SS13	.532		1.4236	.502	.532	.283	.	
**Cues to action (CUE)**								
**Inter-Item Correlation**			**Item-Total Statistics**					Cronbach’s Alpha
Items	CUE2	CUE3	Scale Mean if Item Deleted	Scale Variance if Item Deleted	Corrected Item-Total Correlation	Squared Multiple Correlation	Cronbach’s Alpha if Item Deleted	
CUE2			.2899	.206	.631	.398	.	N/A
CUE3	.631		.3542	.229	.631	.398	.	
**Perceived school support (PSS)**								
**Inter-Item Correlation**			**Item-Total Statistics**					Cronbach’s Alpha
Items	SS9	SS10	Scale Mean if Item Deleted	Scale Variance if Item Deleted	Corrected Item-Total Correlation	Squared Multiple Correlation	Cronbach’s Alpha if Item Deleted	
SS9			2.5226	.514	.599	.358	.	N/A
SS10	.599		2.4010	.557	.599	.358	.	

**Social support (SS)**									
**Inter-Item Correlation**				**Item-Total Statistics**					Cronbach’s Alpha
Items	BAR12	BAR13	BAR14	Scale Mean if Item Deleted	Scale Variance if Item Deleted	Corrected Item-Total Correlation	Squared Multiple Correlation	Cronbach’s Alpha if Item Deleted	
BAR12				.8785	1.992	.487	.238	.829	.778
BAR13	.458			.8524	1.629	.693	.528	.613	
BAR14	.444	.709		.7899	1.554	.678	.521	.628	
**Perceived benefit (PB)**									
**Inter-Item Correlation**				**Item-Total Statistics**					Cronbach’s Alpha
Items	BEN4	BEN6	BEN7	Scale Mean if Item Deleted	Scale Variance if Item Deleted	Corrected Item-Total Correlation	Squared Multiple Correlation	Cronbach’s Alpha if Item Deleted	.755
BEN4				5.4108	1.062	.556	.314	.703	
BEN6	.512			5.4318	1.037	.618	.382	.636	
BEN7	.469	.546		5.4650	.932	.583	.347	.677	
**Perceived school responsibility (PER)**									
**Inter-Item Correlation**				**Item-Total Statistics**					Cronbach’s Alpha
Items	SS11	SS14	SS15	Scale Mean if Item Deleted	Scale Variance if Item Deleted	Corrected Item-Total Correlation	Squared Multiple Correlation	Cronbach’s Alpha if Item Deleted	.721
SS11				4.3872	1.991	.469	.220	.716	
SS14	.420			4.8316	1.566	.585	.358	.579	
SS15	.408	.560		4.3958	1.763	.580	.350	.587	

**Self-efficacy (SE)**											
**Inter-item correlations**						**Item-Total Statistics**					Cronbach’s Alpha
Items	SE1	SE2	SE3	SE4	SE6	Scale Mean if Item Deleted	Scale Variance if Item Deleted	Corrected Item-Total Correlation	Squared Multiple Correlation	Cronbach’s Alpha if Item Deleted	
SE1						9.6233	2.993	.486	.245	.653	.709
SE2	.347					9.4983	3.193	.455	.220	.666	
SE3	.402	.280				9.6319	2.998	.496	.259	.649	
SE4	.302	.282	.358			9.5833	3.287	.431	.191	.675	
SE6	.306	.375	.340	.284		9.8160	3.128	.462	.224	.663	

**Table 5:** Inter-item correlation and item-total statistics of items used to assess adherence to COVID-19 preventive practices among secondary school students in Jimma, Oromia, Ethiopia, 2022.

Items	Inter-item correlations											Item-total statistics					
	P2	P3	P4	P7	P8	P9	P10	P11	P12	P13	P14	Scale Mean if Item Deleted	Scale Variance if Item Deleted	Corrected Item-Total Correlation	Squared Multiple Correlation	Cronbach’s Alpha if Item Deleted	Cronbach’s Alpha
P2												23.8542	21.527	.574	.463	.832	**.849**
P3	.594											23.7378	21.919	.512	.482	.838	
P4	.316	.449										23.3785	23.057	.460	.312	.841	
P7	.329	.319	.327									23.5017	22.546	.537	.431	.836	
P8	.391	.474	.454	.550								23.5330	21.592	.696	.551	.824	
P9	.407	.422	.333	.524	.535							23.6580	21.161	.618	.472	.828	
P10	.420	.236	.167	.172	.389	.336						23.7813	21.549	.515	.394	.838	
P11	.275	.160	.112	.186	.328	.249	.475					23.6493	22.322	.440	.342	.844	
P12	.149	.074	.193	.246	.223	.273	.267	.354				23.3212	23.850	.349	.200	.848	
P13	.375	.287	.300	.371	.472	.507	.413	.275	.232			23.5052	21.534	.587	.402	.831	
P14	.324	.260	.340	.437	.527	.321	.367	.413	.260	.463		23.5069	21.892	.584	.431	.832	

The Pearson correlation coefficient indicated that the extracted factors had positive or negative correlation with each other or practice. For example, percieved social support, perceived school health education and cues to action negatively correlated with perceived school support, perceived benefit, self-efficacy and practice. On the other hand, perceived school health education (PSHE), perceived benefit (PB), perceived school responsibility (PSR), self-efficacy (SE) and attitude to use face mask (AMU) were positively associated with practice [Table 6].

**Table 6:** Pearson correlation coefficient of variables used to determine adherence to COVID-19 self-protective practices among secondary school students in Jimma town, Jimma, Oromia, Ethiopia, 2021.

Inter-item correlation												
Factors	PSS	SS	PS	PSHE	PSEC	Cues to action	PB	PSR	SE	PPI	AMU	Practice
PSS												
SS	-.125											
PS	.097	-.138										
PSHE	-.191	.090	.008									
PSEC	.016	.065	-.097	.050								
Cues to action	-.048	.021	.006	.172	-.088							
PB	.127	-.109	.081	-.104	-.073	-.111						
PSR	.387	-.190	.058	-.268	.244	-.090	.078					
SE	.005	-.055	.097	-.123	-.046	-.158	.369	.119				
PPI	.127	.358	-.176	-.048	-.056	-.049	-.145	-.060	-.040			
AMU	.011	.163	-.072	.074	-.050	.096	.001	.107	-.132	.039		
Practice	-.049	-.029	-.001	-.092	.063	-.134	.331	.154	.381	-.108	.008	

PB: Perceived benefit; SS: Social support; PSS: Perceived school support; SE: Self-efficacy; PSR: Perceived school responsibility; PPI: Perceived peer influence; AMU: Attitude to use face mask; CUE: Cues to action; PS: Perceived severity; PSEC: Perceived school environment cleanness; PSHE: Perceived school health education.

### Item-scale correlations of the deleted items

From a total of thirty extracted items, seven items were deleted because of having low Corrected Item-Total Correlation coefficient. Similarly, three items were deleted from the dependent variable items, i.e., items used to assess adherence to COVID-19 self-protective practices. Attitude towards face mask use (items BAR2, 3 and 4) and perceived peer influence (Items BAR8, 9 and 10) were the two extracted factors but not internally consistent [Table 7].

**Table 7:** Item-total statistics of deleted items used to determine or assess secondary school student’s adherence towards COVID-19 preventive practices in Jimma, Oromia, Ethiopia, 2021.

Items	Scale Mean if Item Deleted	Scale Variance if Item Deleted	Corrected Item-Total Correlation	Squared Multiple Correlation	Cronbach’s Alpha if Item Deleted
SS5: Visual cues (floor markings, posters, etc.) are in place to promote physical distancing	4.9236	1.712	.366	.136	.749
BAR4: I don’t know how to wear a face mask	.8182	1.592	.327	.113	.618
BAR2: Wearing facemask is unnecessary	.4219	.582	.449	.202	.
BAR5: Wearing a face mask makes me look unattractive	.3906	.513	.449	.202	.
P6: Used face masks in classroom	29.9896	27.391	-.346	.253	.793
P1: Shared cups, food or drinks with others students (reversed)	28.0122	27.866	-.132	.213	.829
P5: Avoided going to crowded places in schools such as sports, student gatherings	25.9427	26.325	.102	.076	.849
BAR8: I cannot stop shaking hands because my relationships with people become affected	1.6824	2.409	.409	.174	.590
BAR9: I cannot keep physical distancing because my school is crowed	1.4712	1.928	.501	.251	.457
BAR10: I would feel disappointed by my friends for wearing facemask	1.2094	2.012	.436	.198	.556

#### Predictive validity

A Linear regression analysis indicated that of the extracted factors, only PB (perceived benefit), PSR (perceived school responsibility) and self-efficacy had positively significant association with the COVID-19 self-protective practices [Table 8].

**Table 8:** Association between extracted factors and COVID-19 self-protective practice among secondary school students, Jimma, Oromia, Ethiopia, 2021 (n = 576).

Coefficients							
Model	Unstandardized Coefficients		Standardized Coefficients	t	Sig.	95.0% Confidence Interval for B	
	B	Std. Error	Beta			Lower Bound	Upper Bound
(Constant)	16.369	1.765		9.272	.000	12.901	19.837
PSHE	-.014	.148	-.004	-.097	.923	-.305	.277
Cues	-.367	.231	-.061	-1.591	.112	-.820	.086
PB	.718	.144	.203	4.980	**.000**	.435	1.002
PSR	.255	.106	.095	2.419	**.016**	.048	.463
SE	.669	.097	.282	6.919	**.000**	.479	.859
PPI	-.165	.096	-.065	-1.720	.086	-.353	.023

## Discussion

Factor analysis is a significant instrument which is utilized in development, refinement, and evaluation of tests, scales, and measures. Thus, this study tied to ensure validity and reliability of survey tool used to assess secondary school student’s adherence to COVID-19 preventive practices using EFA and CFA. First, CFA was tried to test the constructs used in the study to identify the factor structure intended to measure given that the study utilized constructs developed based on HBM. However, there was difficulties in fitting the model using established constructs, and too many modifications were needed to fit the model. Therefore, the EFA was carriedout to re-determine and re-name the existing constructs creating new constructs that would measure the variable/construct. Furthermore, the CFA was also tried after running the EFA and creating new factors, but still the model needed too many modifications to be fixed [14–18,25–32].

Accordingly, from the EFA, the study extracted eleven factors (i.e., perceived benefit, perceived school support, social support, self-efficacy, perceived school environment cleanness, perceived school responsibility, perceived school health education, attitude to use face mask, perceived severity, cues to action and perceived peer influence. None of the perceived vulnerability items were extracted during the analysis or not internally consistent. This indicates that the items were not reliable and were not included during principal component analysis given that non-reliable items couldn’t be valid since they would not provide a good estimate of the ability of what should be measured [33–36]. This finding might imply that the students risk perception towards COVID-19 infection was in question. This underscores the need to design appropriate health education program and conduct social and behavioral change communication interventions.

The exploratory factor analysis explored a total of thirty items for the independent variables which were loaded under eleven factors. The final KMO was 0.678 with the Bartlett’s Test of Sphericity (X^2^ = 5697.432; df = 435 and p<0.001). The findings indicated that the study utilized adequate sample size that is enough to run the factor analysis. This is because existing evidences indicated established sample size adequacy to run factor analysis at the KMO cut-off point is 0.50 or greater with Bartlett’s test of Sphericity significance level at p-value <0.05 [15,37].

The extracted items had communality coefficient, proportion of variance explained by the common factors, ranging from 0.464 to 0.722. This means that the items had high values indicating factor solutions explained a sizeable degree of variance for a particular variable or set of variables. Similarly, existing literatures indicated that the item communality coefficient is acceptable when it is greater than 0.40 [33]. The extracted items indicated a cumulative variance of 56.719%. This implies that it had an acceptable level of variance as the minimum requirement is to explain a cumulative variance 50%. Similarly, the items had higher factor loading within range of 0.479 to 0.855. Similarly, this finding showed that the extracted items had fair to excellent loading coefficient as the cut-off point is above 0.45, indicating that the items were valid [38].

Reliability testing was also assessed for extracted factors. For those factors which had two items only correlation coefficient was estimated as it is not possible to estimate the Cronbach’s alpha vale. However, crobach’s alpha was calculated for the rest of the factors. It was found in the range of 0.709 and 0.778. The self-protective items also had overall cronbach’s alpha value 0.849. This indicated that the items described about more than 70% of true individual difference and the rest due to random residual. This implies that the items measuring each construct are internally consistent or reliable as it fulfills the lower cutoff point 0.70 [33,39]. However, items of two extracted factors (i.e., attitude towards face mask use and perceived peer influence) were not internally consistent.

The corrected item-total correlation coefficient on the extracted items was greater than 0.30. ranged from 0.313 to 662. This indicates that there is a strong, positive correlation between the scores on the item values to adequately measure the construct and ensure validity. This implies that the items are internally consistent and meet the criteria to validate the instrument [33,39].

Finally, the predictive validity analysis indicated that only perceived benefit, self-efficacy and perceived school responsibility significantly predicted student’s adherence to COVID-19 self-protective practices. This implies that the survey instrument accurately predicted the outcome of interest. Similarly, existing literatures suggested that the survey is predictively valid if the test accurately predicts what it is supposed to predict/future performance. Therefore, the extracted items are valid and had the ability to predict students’ adherence to COVID-19 self-protective measures [36,37,39].

### Strengths and limitations of the study

To the best of author’s knowledge, this is the first study described and validated a survey instrument used to assess secondary school students adherence to novel COVD-19 in Ethiopia. This would contributes its own impute for policymakers as well as for other researchers on the controlling of the distribution of this pandemic. As a limitation, the study instrument was developed based on the constructs of theory of health belief model and CFA need to be performed. However, since a lot of modification was needed to fit the model, a need raised to run exploratory factor analysis using principal axis factoring to reduce dimensions and extract factors. Second, during development of the scale, experts were consulted to ensure content validity. However, the EFA segmented the previously developed factors and created new factors which might compromise the content validity. Third, there might be a social desirability bias plus bias from self-report. As the study used three-point response format for the questionarrie, there was a need to condduct sensitivity analysis using polychoric correlations, but failed to do it.

## Conclusions

The study found that perceived benefit, perceived school support, social support, self-efficacy, perceived school environment cleanness, perceived school responsibility, perceived school health education, attitude to use face mask, perceived severity, cues to action and perceived peer influence were valid. Perceived vulnerability was neither valid nor reliable construct. Similarly, from the extracted factors, attitude towards face mask use and perceived peer influence were not internally consistent. However, perceived social support, perceived benefit, perceived school responsibility, self-efficacy, and practice items were internally consistent. Finally, perceived benefit, self-efficacy and perceived school responsibility significantly predicted student’s adherence to COVID-19 self-protective practices. Therefore, for better implementation of COVID 19 prevention measures at secondary schools and enhance student’s adherence to self-preventive safety measures, the findings would be useful for concerned bodies to take into consideration. Furthermore, the findings of the study would be helpful for researchers, and also academicians, especially health promotion and communication discipline to develop an appropriate social and behavioral change communication materials targeting to secondary school students.

## Supporting information

S1 File Description of items generated during tool development. (DOCX)

## Abbreviations

CD: Coefficient of determination
CFI: Comparative fit index
COVID-19: Corona virus 19
E.C: Ethiopian calendar
HBM: Health Belief Model
KMO: Kaiser-Meyer-Olkin
PCA: Principal Component Analysis
RMSEA: Root mean squared error approximation
SD: Standard deviation
SRMSR: Standardized root mean squared residual
SPSS: Statistical software for social science
TLI: Tucker-Lewis index
WHO: World Health Organization
