# Supplementary material for: Development and validation of Health Belief Model based instrument to assess secondary school student’s adherence to COVID-19 self-protective practices in Jimma, Oromia, Ethiopia
Source: PLoS One. 2022 Dec 21;17(12):e0279440. doi: 10.1371/journal.pone.0279440 (PMC9770370; doi:10.1371/journal.pone.0279440)
Supplement: S1 File — (DOCX) [file pone.0279440.s001.docx]

| **Self-protective practice items** |
| --- |
| 1. Wash hands frequently with soap and water or uses an alcohol-based hand rub |
| 1. Avoided touching eyes, nose, and mouth before washing hands |
| 1. Avoided shaking hands for the greetings |
| 1. Covered cough using the bend of the elbow or a tissue |
| 1. Shared cups, food, or drinks with other students |
| 1. Maintained physical distancing of at least 1 meters while in the classroom |
| 1. Maintained physical distancing of at least 2 meters while outside the classroom |
| 1. Used facemasks in transportation such as school buses |
| 1. Avoided going to crowded places in schools such as sports, student gatherings |
| 1. Used face masks in the classroom |
| 1. Seat alone on one seat in classroom |
| 1. Staying at home when you were sick or had a common cold or flue |
| 1. Carefully disposing of tissue and disposable items in a closed bin |
| 1. Share what they learn about preventing COVID-19 with family and friends |
| **Perceived vulnerability** |
| 1. No matter what I do, I’m likely to get COVID at school |
| 1. In many aspects, I am less likely to acquire COVID-19 at school |
| 1. Do think you will get a COVID-19 infection at school? |
| 1. Do you think you are at risk for getting COVID-19 because you are a school-going student? |
| 1. Do you think it is less likely to acquire COVID-19 as you are young? |
| 1. I think that there is no COVID-19 disease |
| 1. Only old people are susceptible to COVID-19 |
| **Perceived severity** |
| 1. COVID-19 has had a serious impact on my school performance |
| 1. Do you believe that COVID-19 infection is a severe disease? |
| 1. Do you think that COVID-19 is a dangerous disease? |
| 1. Do you believe that COVID-19 is an extremely harmful disease to your family? |
| 1. Do you think that COVID-19 is a severe disease for young people like you? |
| 1. I am afraid of COVID-19 because people may discriminate me if I get it |
| 1. I don’t care about this disease and I attend my school activities like before |
| **Perceived barriers** |
| 1. It is difficult to find water and soap at school |
| 1. Wearing a facemask is unnecessary |
| 1. It is difficult to get hand sanitizer in school. |
| 1. I don’t know how to wear a face mask |
| 1. Wearing a face mask makes me look unattractive |
| 1. Face mask is uncomfortable to wear, and cause suffocations |
| 1. My family cannot afford to provide me with face mask regularly |
| 1. I cannot stop shaking hands because my relationships with people become affected |
| 1. I cannot keep physical distancing because my school is crowed |
| 1. I would feel disappointed by my friends for wearing a face mask |
| 1. There is no anyone motivates me to wear a face mask |
| 1. There is no anyone motivates me to wash my hands regularly. |
| 1. There is no anyone motivate me to keep physical distance |
| **Perceived benefits** |
| 1. I believe that hand washing is helpful for me to prevent myself from COVID-19 |
| 1. I believe that social distancing is helpful for me to prevent myself from COVID-19 |
| 1. I believe face mask prevents me from getting COVID-19 infections |
| 1. When I use a face mask, I feel a sense of responsibility to protect my families and communities |
| 1. Face mask use is helpful to protect others from the virus |
| 1. I believe that avoiding overcrowding places is helpful for me to prevent myself from COVID-19 |
| 1. I believe that stop shaking people’s hand is helpful for me to prevent myself from COVID-19 |
| 1. I trust the messages my government provides about the pandemic |
| 1. I believe that washing my hands after coughing or sneezing, or doing something is helpful to cure myself and my family. |
| **Self-efficiency** |
| 1. How much you are confident in washing hands frequently with soap and water or using alcohol-based hand rub kills the virus that causes COVID-19 |
| 1. How much you are confident that maintaining social distancing can prevent infection with coronavirus? |
| 1. How much you are confident in avoiding touching eyes, nose, and mouth to prevent infection with coronavirus? |
| 1. How much you are confident in covering your cough/sneezing, using the bend of your elbow or a tissue to prevent the spread of coronavirus? |
| 1. How much you are confident to seek for fever, cough, and difficulty breathing, seeking medical care early help to manage COVID-19? |
| 1. I can maintain at least a 1-meter distance between yourself and another student to prevent infection with coronavirus in school |
| 1. How much you are confident that you can always use a face mask while going to school. |
| **Cues to action** |
| 1. Have you ever seen a person who gets sick from the coronavirus? |
| 1. Did any of your relatives or family members acquire the corona virus? |
| 1. Do your parents remind you how to protect yourself from the coronavirus while you go to school? |
| 1. Received education at school about prevention of COVD-19 |
| **Perceived school support** |
| 1. Students are crowded when they enter and leave the school (reversed) |
| 1. Staff and students can move through common spaces without crowding or physical contact. |
| 1. Physical contact and close, face-to-face interactions are minimized students are spread out as much as possible |
| 1. Physical distancing is practiced by students |
| 1. Visual cues (floor markings, posters, etc.) are in place to promote physical distancing |
| 1. Student gatherings (e.g. events that bring staff and students together outside of regular learning activities) are avoided |
| 1. There is no health school club work on COVID-19 (reversed) |
| 1. Hand cleaning facilities are available and accessible throughout the school and well maintained |
| 1. Signs to remind students to practice regular hand hygiene and good cough etiquette |
| 1. Learning spaces are arranged to maximize the space available and to minimize people directly facing one another |
| 1. My school gives attention to the practice of precautionary measures for the COVID-19 pandemic in the school |
| 1. General cleaning and disinfecting is done every day |
| 1. The school’s ventilation system is serviced and operating w |
| 1. There are an active daily Health Check for students |
| 1. Parents and students are made aware of their responsibilities in COVID prevention |
| 1. Students are reminded to stay home when they are sick |
| 1. Staff wear masks when conducts classroom and outside classroom |
| 1. Masks are available for those who have forgotten theirs |
| 1. There is educational material at school to guide students practice COVID-19 preventive measures |
| 1. There is health education at school on COVID-19 preventive measures |
